# Flexure and shear response of an impulsively loaded rigid-plastic beam by enhanced linear complementarity approach

**DOI:** 10.1038/s41598-022-14082-4

**Published:** 2022-06-14

**Authors:** Azam Khan, Moiz Tariq, Asad Ullah, Niaz B. Khan, Mohammed Jameel

**Affiliations:** 1 National University of Sciences and Technology (NUST), Islamabad, Pakistan; 2 Department of Civil Engineering, College of Engineering, King Khalid University, Abha, Saudi Arabia

**Keywords:** Mechanical properties, Civil engineering

## Abstract

The linear complementarity approach has been utilized as a systematic and unified numerical process for determining the response of a rigid-plastic structure subjected to impulsive loading. However, the popular Lemke Algorithm for solving linear complementarity problems (LCP) encounters numerical instability issues whilst tracing the response of structures under extreme dynamic loading. This paper presents an efficient LCP approach with an enhanced initiation subroutine for resolving the numerical difficulties of the solver. The numerical response of the impulsively loaded structures is affected by the initial velocity profile, which if not found correctly can undermine the overall response. In the current study, the initial velocity profile is determined by a Linear Programming (LP) subroutine minimizing the energy function. An example of a uniform impulsively loaded simply supported beam is adduced to show the validity and accuracy of the proposed approach. The beam is approximated with bending hinges having infinite resistance to shear. Comparison of the numerical results to the available closed-form solution confirms the excellent performance of the approach. However, a subsequent investigation into a beam having the same support conditions and the applied loading, but with bending and shear deformation, results in numerical instability despite optimizing the initial velocity profile. Thus a more generic description of kinetics and kinematics is proposed that can further enhance the numerical efficiency of the LCP formulation. The ensuing numerical results are compared with the available close form solution to assess the accuracy and efficiency of the developed approach.

## Introduction

Blast-protective structures find wide application in nuclear power plants, oil refineries, ship and aircraft industries, bridges, etc. Due to explosives or impact loading, these structures respond in a manner that is complex and nonlinear in nature^1,2^. Thus extensive experimental, analytical and numerical investigations have been carried out to study the mitigative effect of the protective structures under extreme loads^3–8^.The rapid advancement of computers has motivated various analysis techniques capable to model the complex dynamic response to a high level of accuracy^9–11^.

Many simplified approaches exist in the literature to predict the response of structures under extreme dynamic loading. Generally, analytical models based on a single degree of freedom or two degrees of freedom can be used to estimate the response parameters, e.g. the maximum displacement. The advantage of these models is that they can be easily codified in a programming language Nevertheless, such approaches can only be used as a first approximation of the actual dynamic response of structures leading to underestimation of various response parameters^9–11^. For accurate determination of the dynamic response of these structures under extreme loading, commercially available software packages such as ABAQUS, ANSYS, LS-DYNA are most popularly adopted. However, developing accurate simulations can result in a significant computation cost, especially when assessing large skeletal structures^9–11^.

Mathematical programming is seemingly one of the most natural formalisms in which to cast the skeletal structures for determining the extreme dynamic response^12^. It readily offers an ideal framework capable of finite element modeling of structures, and also provides considerable physical insights into the dynamic behavior of structures^13–18^. For the past few decades, mathematical programming has exhibited its virtues in various engineering applications, as propounded by Maier and Munro^19^, Maier^20^, and Maier and Lloyd Smith^21^.

Quadratic programming is a useful computational device ideal for representing discretely rigid-plastic skeletal structures under impulsive loading^22–25^. An application of this formulation to layout optimization of impulsively loaded rigid plastic structures is presented by Kaliszky and Logo^26^. A further extension of the quadratic program has been rigid plastic modeling of walls subjected to extreme dynamic loading^27^. Essentially, the concept of mathematical programming has been successfully employed by Smith and Sahlit^28,29^ and Khan et al.^30^.

There seems to be considerable interest in the study of rigid-plastic structures under extreme dynamics loading as is reflected by the large body of work in the area; Lee and Symonds^31^, Symonds^32^, and Jones^33,34^. The idea of utilizing rigid-plastic idealization was suggested by Lee and Symonds^31^ while investigating the response of free-free beam subject to blast loading. Later, Parkes^27^ devised the concept of the traveling plastic hinge, thus motivating series of inspired works^35,36^. More recently, this simple theory has been extended to incorporate the effects of the bending-shear interactions, strain rate and large displacements^37–43^.

The effect of shear forces appears to be important in evaluating the quasit-static behavior of structures^43–46^, but more significant in the structures submitted to extreme dynamic loading, Bleich and Shaw^47^. The shear force effect can markedly influence the behavior of those beams in which the dynamic response involves higher modes, Symonds^48,49^ and Jones^50^. It transpires that the shear effects are more important for the I beams than for the rectangular beams of the same length; Menkes and Opat^51^, Jones^52^ and Karunes and Onat^53^. Regardless of the properties of any cross-section, the interaction curve relating the shear force and the bending moment cannot be uniquely defined. Mathematical convenience has motivated researchers to approximate the interaction relation to rectangular yield criterion, Symonds^48,49,54^. This approach has lead to the notion of shear sliding within the sheared zone is commonly adopted^50,52,55–59^, which can be conveniently modeled by 1D rigid-plastic solid elements^30^.

A detailed 1D rigid-plastic modeling strategy has been developed previously^25,30^ which was validated against the continuum solution of the impacted beam. This numerical approach, formulated as a Linear Complementarity Problem (LCP), led to fast and accurate dynamic response prediction, unlike the commercial finite element softwares that require extensive computer memory and a longer execution time^60^. However, there were instances when the accuracy of the LCP solver^25^ was undermined by issues concerning numerical instability. These problems were attributed to the approximate initial velocity profile, which is required to initiate the numerical process.

To achieve the numerical stability of the LCP approach, the current study proposes a new initiation technique that utilizes a linear program (LP) to determine the actual initial velocity profile. Lumped mass and continuous mass elements are employed to model simply supported beam having infinite resistance to shear deformation. The beam is subjected to impulsive loading and the initial velocity profile is determined from the newly developed LP, which is implemented in the LCP approach^30^. The accuracy and numerical efficiency of the results are confirmed by comparing the results with the continuum solution of the beam problem. However, the numerical difficulties reappear in the subsequent investigation of the impulsively loaded beam in which shear deformation is allowed at the support location. An improved kinetic and kinematic description is developed to circumvent these difficulties. Results from the shear critical simply supported beam ensure the computational robustness of the LCP using this general description.

## Improved dynamic rigid-plastic model

In the previous research^30^, the formulation in the form of a Linear Complementarity Problem (LCP) has been developed to determine the response of rigid-plastic beams under impact loading. The initiation sub-routine of that formulation is modified herein to resolve the numerical difficulties that appeared previously. Besides, the formulation is extended to beams under impulsive loading, which shows pronounced sensitivity with the current semi-definite LCP^28,29^.

### Representation of kinetics and kinematics as nodal description

**Figure 1:**
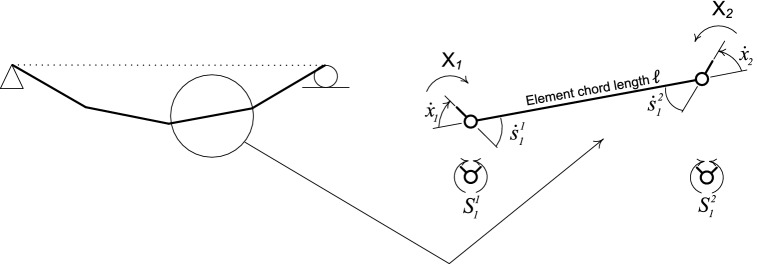
Stress-resultants, strain-resultant rates, chord deformation rates and independent chord forces in a element of a discretized member.

The kinetic and kinematic setting of the actual continuous structure of Fig. 1 is revisited herein. This structure is replaced with a system of N discretized regions, whose nodal movements are controlled by the β degrees of freedom. Considering α static indeterminacy of the structure and S plastic rotational deformation at the nodes, the kinematic indeterminacy number takes the form $\beta =S-\alpha$.

**Figure 2:**
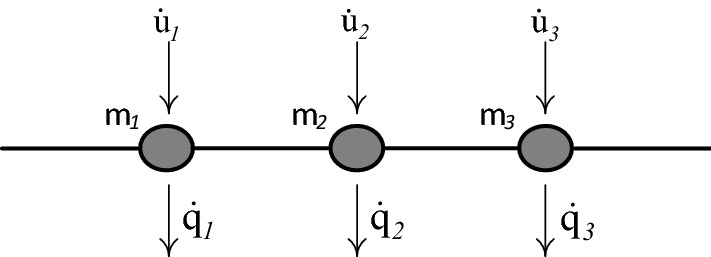
Lumped-mass system having centroidal velocities ${\dot{\mathbf{u}}}$ related to inertia forces and independent nodal velocities ${\dot{\mathbf{q}}}$.

**Figure 3:**
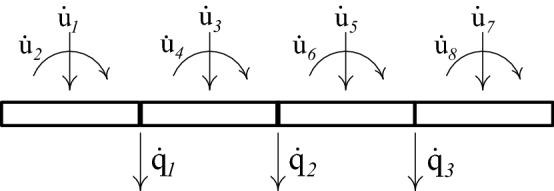
Continuous mass system having centroidal velocities ${\dot{\mathbf{u}}}$ related to inertia forces and independent nodal velocities ${\dot{\mathbf{q}}}$.

**Figure 4:**
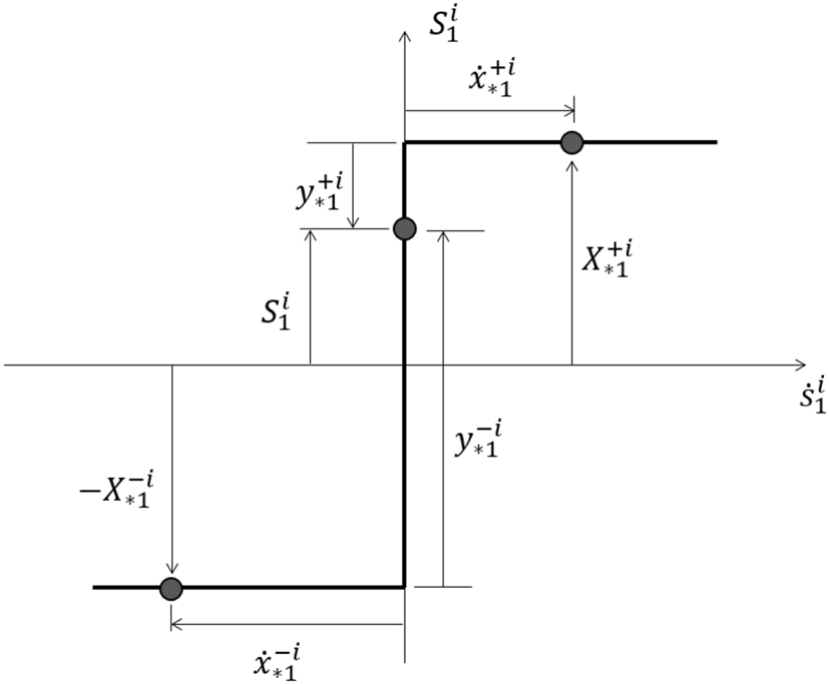
Material characteristics of rigid-plastic behavior indicating plastic potentials ${\mathbf{y}}_{*}$ and plastic multiplier rates ${\dot{\mathbf{x}}}_{\user2{*}}$.

It can be shown that, when β independent nodal velocities ${\dot{q}}_{j } \left(j=\mathrm{1,2},\dots \dots ,\beta \right)$ are released, there exists a kinematically consistent independent member deformation rates ${\dot{x}}_{h }\left(h =1, 2, \dots .., 2N\right)$; indicated in Fig. 2, velocities related to center of gravity of mass ${\dot{u}}_{k}\left(k =1, 2, \dots .., \gamma \right)$; indicated in Figs. 3 and 4, and load point velocities ${\dot{\delta }}_{\mathcal{l}}\left(\mathcal{l}=1, 2, \dots .., n\right)$.

Hence, the nodal setting of kinematics must be written:

1 $$\left[ {\begin{array}{*{20}c} {{\dot{\mathbf{x}}}} \\ {{\dot{\mathbf{u}}}} \\ {{\dot{\mathbf{\delta }}}} \\ \end{array} } \right] = \left[ {\begin{array}{*{20}c} {\mathbf{A}} \\ {{\mathbf{A}}_{d} } \\ {{\mathbf{A}}_{0} } \\ \end{array} } \right]{\dot{\mathbf{q}}},$$

where the coefficient matrix is constant, provided that the motion falls within small displacements.

It may be realized that a dual equation must exist which defines the nodal settings of system kinetics. Thus D’ Alembert principle can be applied to a structure having n discrete time-dependent nodal applied loads ${\lambda }_{\mathcal{l}}\left(\mathcal{l}=1, 2, \dots .., n\right)$, which invokes the inertia forces ${\mu }_{k}\left(k =1, 2, \dots .., \gamma \right)$ and the independent member forces ${X}_{h }\left(h =1, 2, \dots .., 2N\right)$. Similar to the independent nodal displacements, the nodal forces of constraint ${Q}_{j}\left(j =1, 2, \dots .., \beta \right)$ must vanish, thereby resulting in the kinetic equation

2 $${\mathbf{Q}} = { }0 = { }\left[ {\begin{array}{*{20}c} {{\mathbf{A}}^{T} } & {{\mathbf{A}}_{d}^{T} } & {{\mathbf{A}}_{0}^{T} } \\ \end{array} } \right]\left[ {\begin{array}{*{20}c} {\mathbf{X}} \\ { - {{\varvec{\upmu}}}} \\ { - {{\varvec{\uplambda}}}} \\ \end{array} } \right],$$

where the transposed (T) coefficient matrix remains constant by the virtue of small displacements.

The inertia forces ${\mu }_{k}\left(k =1, 2, \dots .., \gamma \right)$ in (2) can be linked to the centroidal accelerations $\ddot{u}_{k} \left( {k = 1, 2, \ldots , \gamma } \right)$

3 $${{\varvec{\upmu}}} = - {\mathbf{m}}\ddot{\mathbf{u}}$$

where m is the diagonal mass matrix $m_{k} \left( {k = 1, 2, \ldots , \gamma } \right)$ constituting the mass or moment of inertia related to the centroidal accelerations.

### Material model

The constitutive relation between the stress-resultant $S_{1}^{i}$ (bending moment $M_{i}$) and the strain-resultant rate $\dot{s}_{1}^{i}$ (rotation rate $\dot{\theta }_{i}$) at the control node section $i \left( {i = 1,2, \ldots , \chi } \right)$ of the discrete model is shown in Fig. 4. The vectors ${\mathbf{y}}_{*}$ and ${\dot{\mathbf{x}}}_{*}$ are the respective plastic potentials and the plastic multiplier rates, both controlling the plastic yielding at the critical section. ${\mathbf{X}}_{*}$ defines the vector containing the plastic capacities of all critical sections. Finally, it must be noted that the plastic potentials, the plastic multiplier rates, and the plastic capacities are non-negative at $+i$ or $-i$ direction.

In view of Fig. 4 and the above description, the nonholonomic plasticity relations can be straightforwardly approximated^61^ as

4 $$\left[ {\begin{array}{*{20}c} 0 & {{\mathbf{N}}^{{\varvec{T}}} } \\ {\mathbf{N}} & 0 \\ \end{array} } \right]\left[ {\begin{array}{*{20}c} {{\dot{\mathbf{x}}}_{{*}} } \\ {\mathbf{S}} \\ \end{array} } \right] + \left[ {\begin{array}{*{20}c} {{\mathbf{y}}_{{*}} } \\ 0 \\ \end{array} } \right] = \left[ {\begin{array}{*{20}c} {{\mathbf{X}}_{{*}} } \\ {{\dot{\mathbf{s}}}} \\ \end{array} } \right]$$

5, 6, 7 $${\mathbf{y}}_{*} \ge 0\quad {\mathbf{y}}_{*}^{T} {\dot{\mathbf{x}}}_{{*}} = 0 \quad {\dot{\mathbf{x}}}_{*} \ge 0$$

where **N** is the vector of identity matrix $\mathbf{I}$ in the form $$\left[\begin{array}{cc}\mathbf{I}& -\mathbf{I}\end{array}\right]$$.

To develop a governing mathematical system, it is necessary to provide the following transformations that link the independent member forces **X** and the stress-resultants **S**, and their dual deformation rates $\dot{\mathbf{x}}$ and strain resultants $\dot{\mathbf{s}}$:

8,9 $${\dot{\mathbf{x}}} = {\mathbf{T}}\dot{\mathbf{s}},~\quad {\mathbf{S}} = {\text{~}}{\mathbf{T}}^{T} {\mathbf{X}}.$$

### The mathematical formulation

Before developing a governing system based upon the foregoing relations, it is essential to select an approximate time-integration scheme that can determine a numerical solution sequentially. Hence the independent nodal velocities and the nodal displacements are advanced from time $t_{n}$. to $t_{n + 1}$ using Newmark time integration scheme, that is,

10a $${\dot{\mathbf{q}}}_{{{\varvec{n}} + 1}} = {\dot{\mathbf{q}}}_{{\varvec{n}}} + \left( {1 - {\overline{\mathbf{\gamma }}}} \right)\Delta {\mathbf{t}}\ddot{\mathbf{q}}_{{\varvec{n}}} + {\overline{\mathbf{\gamma }}}\Delta {\varvec{t}}{\ddot{\mathbf{q}}}_{{{\varvec{n}} + 1}} ,$$

10b $${\mathbf{q}}_{n + 1} = {\mathbf{q}}_{n} + \Delta t{\dot{\mathbf{q}}}_{n} + \left( {\frac{1}{2} - \overline{\alpha }} \right)\Delta t^{2} {\ddot{\mathbf{q}}}_{n} + \overline{\alpha }\Delta t^{2} {\ddot{\mathbf{q}}}_{n + 1} ,$$

in which $\overline{\alpha }$, $\overline{\gamma }$ are the integration constant affecting the stability and the accuracy of the numerical process. By rewriting (10a)

11 $${\ddot{\mathbf{q}}}_{n + 1} = b_{0} \left( {{\dot{\mathbf{q}}}_{n + 1} - {\dot{\mathbf{q}}}_{n} } \right) - b_{1} {\ddot{\mathbf{q}}}_{n} ,$$

where the integration constants are:

12,13 $$b_{0} = \frac{1}{{\overline{\gamma }\Delta t}} ,\quad b_{1} = \frac{{1 - \overline{\gamma }}}{{\overline{\gamma }}}$$

Stability and robustness of rigid plastic dynamics seem to be ensured when the average acceleration scheme is constant, in which $\overline{\alpha }=0.25$ and $\overline{\gamma }=0.5$.

Considering Eqs. (1) to (9) at time $t={t}_{n+1}$ , and introducing Newmark scheme (11) to (13) the governing system can be written in the form

14 $$\left[ {\begin{array}{*{20}l} { - b_{0} {\mathbf{M}}_{q} } \hfill & { - {\mathbf{A}}^{T} } \hfill & {\mathbf{0}} \hfill & {\mathbf{0}} \hfill \\ { - {\mathbf{A}}} \hfill & {\mathbf{0}} \hfill & {{\mathbf{TN}}} \hfill & {\mathbf{0}} \hfill \\ {\mathbf{0}} \hfill & {{\mathbf{N}}^{T} {\mathbf{T}}^{T} } \hfill & {\mathbf{0}} \hfill & {\mathbf{I}} \hfill \\ \end{array} } \right]\left[ {\begin{array}{*{20}l} {{\dot{\mathbf{q}}}_{{n + {1}}} } \hfill \\ {{\mathbf{X}}_{{n + {1}}} } \hfill \\ {{\dot{\mathbf{x}}}_{{*n + {1}}} } \hfill \\ {{\mathbf{y}}_{{*n + {1}}} } \hfill \\ \end{array} } \right] = \left[ {\begin{array}{*{20}c} { - {\mathbf{Y}}_{{n + {1}}} } \\ {\mathbf{0}} \\ {{\mathbf{X}}_{*} } \\ \end{array} } \right]$$

15,16,17 $${\mathbf{y}}_{{{*}n + 1}} \ge 0\quad {\mathbf{y}}_{{n + 1}}^{T} {\dot{\mathbf{x}}}_{{{*}n + 1}} = 0 \quad {\dot{\mathbf{x}}}_{{{*}n + 1}} \ge 0$$

The right-hand side sub-vector ${\mathbf{Y}}_{n+1}$ of (14) and the mass matrix ${\mathbf{M}}_{{\varvec{q}}}$ are given by:

18,19 $${\mathbf{Y}}_{{{\varvec{n}} + 1}} = {\mathbf{A}}_{0}^{{\varvec{T}}} {{\varvec{\uplambda}}}_{{{\varvec{n}} + 1}} + {\mathbf{M}}_{{\varvec{q}}} \left( {{\varvec{b}}_{0} {\dot{\mathbf{q}}}_{{\varvec{n}}} + {\varvec{b}}_{1} {\ddot{\mathbf{q}}}_{{{\varvec{n}} + 1}} } \right),{\mathbf{M}}_{{\varvec{q}}} = {\mathbf{A}}_{{\varvec{d}}}^{{\varvec{T}}} {\mathbf{mA}}_{{\varvec{d}}} ,$$

The formalism (14) to (17) has a mathematical structure of a linear complementarity problem (LCP). It is important to appreciate that the velocities ${\dot{\mathbf{q}}}_{n+1}$ for the next iteration are given directly, the accelerations ${\ddot{\mathbf{q}}}_{n+1}$ should not be found from (11) since it does not satisfy the kinetic law (2). Instead, by continuing to satisfy that law, together with (1) and (3),

20 $${\mathbf{M}}_{{\varvec{q}}}{\ddot{\mathbf{q}}}_{{\varvec{n}}+1}=\boldsymbol{ }{\mathbf{A}}_{0}^{{\varvec{T}}}{{\varvec{\uplambda}}}_{{\varvec{n}}+1}-{\mathbf{A}}^{{\varvec{T}}}{\mathbf{X}}_{{\varvec{n}}+1}$$

This can be solved uniquely for ${\ddot{\mathbf{q}}}_{n+1}$ for appropriate modeling having positive definite ${\mathbf{M}}_{q}$. Of course, for motion caused by the application of all impulses at the same time, $t={t}_{0}$, the load vectors in (18) and (20) are ${{\varvec{\uplambda}}}_{n+1}=0$ .

### Initiation

For the impulsive loading, the LCP formulation (14) to (17) requires a velocity profile to initiate the evolutionary process. Although the initial displacements ${\mathbf{q}}_{0}$ at time $t={t}_{0}$ are directly prescribed, and usually ${\mathbf{q}}_{0}=0$, the initial velocities ${\dot{\mathbf{q}}}_{0}$ must be inferred from the prescribed magnitude and distribution of the initial loading impulses ${\varvec{\Omega}}$. Integrating (2) with respect to time, over the small interval $\left[{t}_{0}-\varepsilon , {t}_{0}+\varepsilon \right]$ containing the impulsive loading, substituting (1) and (3), and taking the limit

21 $$\mathop {\lim }\limits_{{\user2{\varepsilon } \to 0}} \left[ {{\mathbf{A}}^{\user2{T}} \mathop \smallint \limits_{{\user2{t}_{0} - \user2{\varepsilon }}}^{{\user2{t}_{0} + \user2{\varepsilon }}} {\mathbf{X}}\user2{dt} + {\mathbf{M}}_{\user2{q}} \mathop \smallint \limits_{{\user2{t}_{0} - \user2{\varepsilon }}}^{{\user2{t}_{0} + \user2{\varepsilon }}} {\ddot{\mathbf{q}}}\user2{dt} - {\mathbf{A}}_{0}^{\user2{T}} \mathop \smallint \limits_{{\user2{t}_{0} - \user2{\varepsilon }}}^{{\user2{t}_{0} + \user2{\varepsilon }}} {\mathbf{\lambda }}\user2{~dt}} \right] = 0$$

The first integral vanishes because the element end moments $\mathbf{X}$ for all elements are assumed to be bounded by the finite plastic moment values ${\mathbf{X}}_{*}$. The second integral gives the velocities ${\dot{\mathbf{q}}}_{0}$ at $t={t}_{0}$ acquired from rest due to the initial impulses ${\varvec{\Omega}}$, which are given by the third integral. Thus

22 $${\mathbf{M}}_{{\varvec{q}}}{\dot{\mathbf{q}}}_{0}={\mathbf{A}}_{0}^{{\varvec{T}}}{\varvec{\Omega}}$$

which allows ${\dot{\mathbf{q}}}_{0}$ to be solved uniquely for modeling having positive definite ${\mathbf{M}}_{q}$.

Now consider the evolutive process (14) to (17), which is not self-starting. So a self-starting subroutine is developed which calculates the relevant accelerations in (18). For determining these accelerations, the vector of plastic multiplier rate ${\dot{\mathbf{x}}}_{*}$ is partitioned into the subset Y yielded (active) section and the subset R rigid (non-active) sections as.

23 $$Y = \left\{ {\left( {{\dot{\mathbf{x}}}_{*y} , {\mathbf{y}}_{*y} } \right){|}{\dot{\mathbf{x}}}_{*y} > 0, {\mathbf{y}}_{*y} = 0} \right\},$$

24 $$R = \left\{ {\left( {{\dot{\mathbf{x}}}_{*r} , {\mathbf{y}}_{*r} } \right){|}{\dot{\mathbf{x}}}_{*r} = 0, {\mathbf{y}}_{*r} \ge 0} \right\}$$

The yielded y and rigid r subscripts, shown in (23) and (24), can be employed to derive an acceleration relation of Tamuzh^62^

25 $$\left[ {\begin{array}{*{20}c} {\mathbf{0}} & {\mathbf{0}} & {{\mathbf{N}}_{y}^{T} } \\ {\mathbf{0}} & {\mathbf{0}} & {{\mathbf{N}}_{r}^{T} } \\ {{\mathbf{N}}_{y} } & {{\mathbf{N}}_{r} } & {\mathbf{0}} \\ \end{array} } \right]\left[ {\begin{array}{*{20}c} {{\ddot{\mathbf{x}}}_{*y} } \\ {\ddot{{\mathbf{x}}}}_{*r} \\ {\mathbf{S}} \\ \end{array} } \right] + \left[ {\begin{array}{*{20}c} {\mathbf{0}} \\ {{\mathbf{y}}_{*r} } \\ {\mathbf{0}} \\ \end{array} } \right] = \left[ {\begin{array}{*{20}c} {{\mathbf{X}}_{*y} } \\ {{\mathbf{X}}_{*r} } \\ {{\ddot{\mathbf{s}}}} \\ \end{array} } \right]$$

26,27,28 $${\mathbf{y}}_{{{*}r}} \ge 0\quad {\mathbf{y}}_{{{*}r}}^{T} {\ddot{\mathbf{x}}}_{{{*}r}} = 0\quad {\ddot{\mathbf{x}}}_{{{*}r}} \ge 0$$

29 $${\ddot{\mathbf{x}}}_{*y} \;unrestricted.$$

Thus, in view of (1) and (25) to (29), the governing system^28,29^ of acceleration can be established as

30 $$\left[ {\begin{array}{*{20}c} { - {\mathbf{M}}_{q} } & { - {\mathbf{A}}^{T} } & {\mathbf{0}} & {\mathbf{0}} & {\mathbf{0}} \\ { - {\mathbf{A}}} & {\mathbf{0}} & {{\mathbf{T}}_{y} {\mathbf{N}}_{y} } & {{\mathbf{T}}_{r} {\mathbf{N}}_{r} } & {\mathbf{0}} \\ {\mathbf{0}} & {{\mathbf{N}}_{y}^{T} {\mathbf{T}}_{y}^{T} } & {\mathbf{0}} & {\mathbf{0}} & {\mathbf{0}} \\ {\mathbf{0}} & {{\mathbf{N}}_{r}^{T} {\mathbf{T}}_{r}^{T} } & {\mathbf{0}} & {\mathbf{0}} & {{\mathbf{I}}_{r} } \\ \end{array} } \right]\left[ {\begin{array}{*{20}c} {{\ddot{\mathbf{q}}}} \\ {\mathbf{X}} \\ {{\ddot{\mathbf{x}}}_{*y} } \\ {{\ddot{\mathbf{x}}}_{*r} } \\ {{\mathbf{y}}_{*r} } \\ \end{array} } \right] = \left[ {\begin{array}{*{20}c} { - {\mathbf{A}}_{{0}}^{{\text{T}}} {{\varvec{\uplambda}}}_{{0}} } \\ {\mathbf{0}} \\ {{\mathbf{X}}_{*y} } \\ {{\mathbf{X}}_{*r} } \\ \end{array} } \right]$$

31,32,33 $${\mathbf{y}}_{{{*}r}} \ge 0\quad {\mathbf{y}}_{{{*}r}}^{T} {\ddot{\mathbf{x}}}_{{{*}r}} = 0 \quad {\ddot{\mathbf{x}}}_{{{*}r}} \ge 0$$

If the structure is coerced into motion by an initial impulse or by an initial velocity field, the vector of initial loading is null ${{\varvec{\uplambda}}}_{0}=0$ at the start of motion. Accordingly, the set of $Y$ active plastic hinges $\left({\dot{\mathbf{x}}}_{\boldsymbol{*}{\varvec{y}}}>0\right)$ can be easily deduced.

To establish the initial governing system (30) to (33) of the impulsively loaded structure, the yield surfaces activated by the initial velocities ${\dot{\mathbf{q}}}_{0}$ must be determined. More formally, it follows from (4) to (7), if ${\dot{\mathbf{q}}}_{0}$ is found from (22), ${\dot{\mathbf{x}}}_{*}$ can be defined using the linear program

34 $${\varvec{M}}{\varvec{i}}{\varvec{n}}{\varvec{i}}{\varvec{m}}{\varvec{i}}{\varvec{s}}{\varvec{e}}:\boldsymbol{ }\boldsymbol{ }\mathbf{z}=\boldsymbol{ }{\mathbf{X}}_{\boldsymbol{*}}^{{\varvec{T}}}{\dot{\mathbf{x}}}_{\boldsymbol{*}},\quad \boldsymbol{ }\boldsymbol{ }\boldsymbol{ }\boldsymbol{ }\boldsymbol{ }\boldsymbol{ }\boldsymbol{ }\boldsymbol{ }{\varvec{s}}{\varvec{u}}{\varvec{b}}{\varvec{j}}{\varvec{e}}{\varvec{c}}{\varvec{t}}\boldsymbol{ }{\varvec{t}}{\varvec{o}}:\mathbf{T}\mathbf{N}{\dot{\mathbf{x}}}_{\boldsymbol{*}}=\mathbf{A}\dot{\mathbf{q}},\quad \,\boldsymbol{ }\boldsymbol{ }{\dot{\mathbf{x}}}_{\boldsymbol{*}}\ge 0.$$

Notably, by incorporating the above linear program in a newly developed subroutine, it turns out that most of the numerical difficulties that followed previously^30^ due to the refinement of mesh disappear.

### Plastic unstressing

The evolutive formulation (14) to (17) is unable to determine plastic unstressing within an increment ∆t. In order to sustain the accuracy and stability of the numerical process, another subroutine is included that determines the unstressing time within the interval ∆t^28–30^. Once the unstressing is detected, the evolutionary process is temporarily terminated, the multiplier rates are partitioned into active Y and in-active R sections, and the system (14) to (17) is reinitiated from the unstressing time.

### Impulsively loaded beam with simple supports

**Figure 5:**
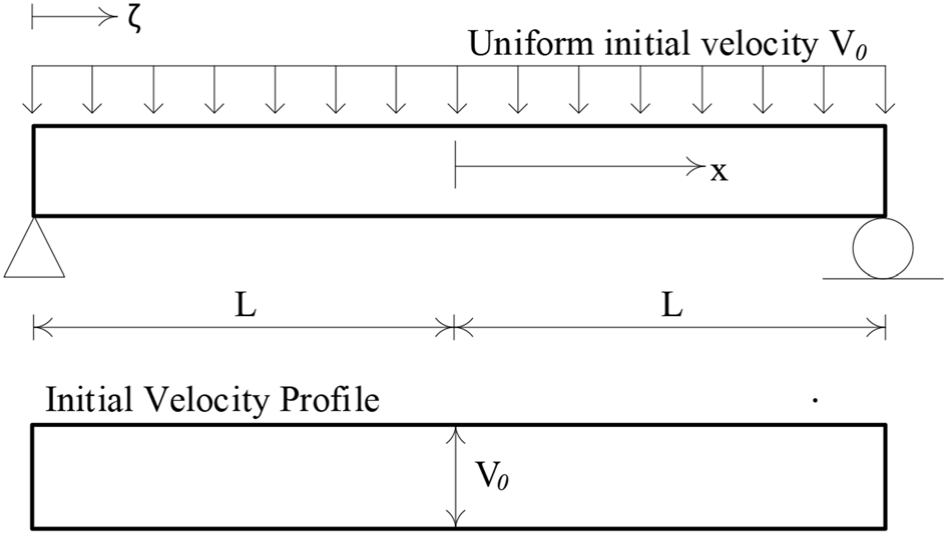
Illustration of impulsively loaded simply supported beam having a rectangular transverse initial velocity profile.

The accuracy and reliability of the proposed LCP formulation implementing the new initiation linear program (34) are best assessed by applying to an example of a simply supported beam under impulsive loading, Fig. 5. The beam is assumed to have infinite resistance to plastic shear deformation. The results of the proposed numerical procedure are compared with the exact solution obtained by Jones^34^.

### Problem statement

Figure 5 shows the beam subjected to the action of a uniformly distributed vertical impulse of total value $I$ which produces a uniform initial transverse instantaneous velocity ${V}_{0}= I/2mL$. Let the length of the beam be $2L$ with uniform mass per unit length $m$ and let the plastic moment capacity be ${M}_{p}$ for bending of either sense in the vertical plane.

### Theoretical beam response

**Figure 6:**
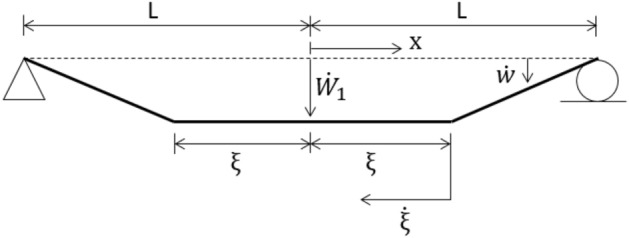
Sketch showing the distribution of transverse velocity during Phase 1 of motion.

**Figure 7:**
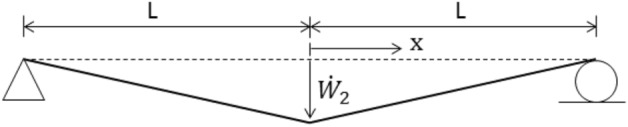
Sketch showing the distribution of transverse velocity during Phase 2 of the motion.

A continuum solution for the rigid-plastic beam of Fig. 5 has been presented by Symonds^63^, and in some detail by Jones^34^. Just after the instant of loading, the cross-sections directly over the supports have no initial velocity and no initial displacement; the rest of the beam acquires an initial velocity ${V}_{0}$ at no initial displacement, Fig. 5. Phase 1 of the motion begins with plastic hinges forming within the beam span and then traveling towards the midspan section. This velocity profile is illustrated in Fig. 6. Phase 2 of the motion initiates when the traveling plastic hinges meet at the midspan and this phase terminates when all the kinetic energy is consumed by the stationary hinge at the midspan. The velocity distribution of Phase 2 is shown in Fig. 7.

A typical requirement of the theoretical solution is the knowledge of the velocity profile during any phase of motion. The proposed linear program (34) identifies and calculates the velocity profile automatically.

### Dynamic rigid-plastic response predicted by LCP solution

The beam of Fig. 5 is discretized into $N$ finite elements of equal length. The evolution of rigid-plastic deformation is investigated using two rigid element types: (a) the lumped-mass element, Fig. 2, and (b) the uniform-mass element, Fig. 3.

**Figure 8:**
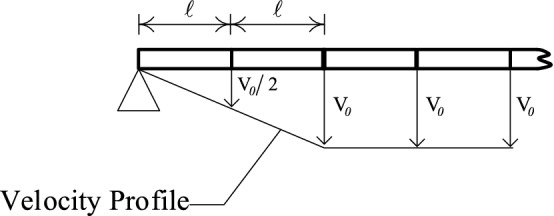
Example of approximate initial velocity profile when the simply supported beam is discretized into uniform mass elements.

To initiate the numerical sequence of LCP formulation, the initial velocity profile is required. In the case of lumped mass elements, this is rectangular shaped having a value of ${V}_{0}= I/2mL$at all the masses, excepting those directly over the supports. In the case of uniform mass element, the vector ${\dot{\mathbf{q}}}_{0}$ of initial velocities is calculated from (22) with a discrete vertical impulse $I/N$ applied at the centroid of each finite element. Given these initial velocities, the linear program (34) generates an optimum velocity profile shown in Fig. 8, which the LCP solver can handle very effectively.

**Figure 9:**
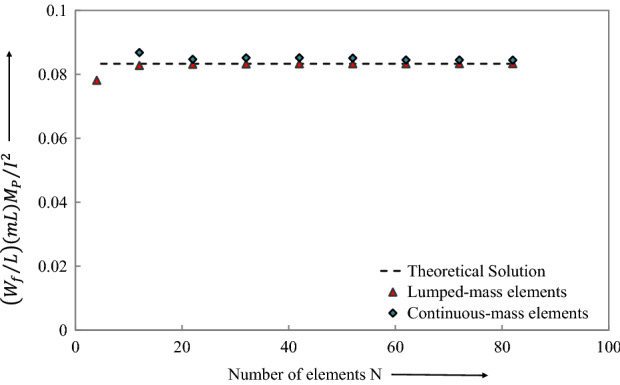
Final central displacement of the beam predicted by theoretical and LCP discretizations.

By examining the results offered by LCP obtained for both lumped-mass elements (Table 1) and uniform-mass elements (Table 2), it is clear that the lumped -mass element presented a very small error. These tables present the results for the non-dimensional central displacement ${\overline{W} }_{f}=\left({W}_{f}/L\right)(mL){M}_{p}/{I}^{2}$ and the non-dimensional cessation time $\overline{t }={M}_{p}{t}_{1}/IL$ for a range of finite elements. Likewise, the range of finite elements is employed to achieve convergence, Fig. 9.

**Table 1:** Comparison of the theoretical solution of Norman Jones^34^ with LCP solution for the case of lumped-mass elements.

Error in displacement (%)	End time of phase 1 $\overline{t} = M_{p} t_{1} /IL$	Error in phase 1 end time (%)
0.0164	0.083283	0.060
0.019	0.083269	0.077
0.025	0.083247	0.103
0.037	0.083210	0.148
0.056	0.083145	0.226
0.098	0.083008	0.390
0.207	0.082645	0.827
0.695	0.081018	2.778

**Table 2:** Comparison of theoretical solution of Norman Jones^34^ with LCP solution for the case of uniform-mass elements.

No of elements $N$	Final central displacement $\overline{W}_{f} = \left( {W_{f} /L} \right)\left( {mL} \right)M_{p} /I^{2}$	Error in displacement (%)	End time of phase 1 $\overline{t} = M_{p} t_{1} /IL$	Error in phase 1 end time (%)
112	0.084365456	1.223	0.079883452	4.139
82	0.084460884	1.335	0.078928571	5.286
72	0.084490103	1.369	0.078321429	6.014
62	0.084497373	1.377	0.077500000	7.000
52	0.085102077	2.078	0.077017857	7.579
42	0.085182345	2.171	0.075571429	9.314
32	0.085163257	2.149	0.073206983	12.152
22	0.084707255	1.622	0.068632369	17.641
12	0.086805191	4.000	0.061442415	26.270

### Theoretical solution (Jones^34^):

Final Central Displacement ${\overline{W} }_{f}=\left({W}_{f}/L\right)(mL){M}_{p}/{I}^{2}=0.08\dot{3}$

Phase 1 Cessation time $\overline{t }={M}_{p}{t}_{1}/IL=0.08\dot{3}$

### Theoretical solution (Jones^34^):

Final Central Displacement ${\overline{W} }_{f}=\left({W}_{f}/L\right)(mL){M}_{p}/{I}^{2}=0.08\dot{3}$

Phase 1 Cessation time $\overline{t }={M}_{p}{t}_{1}/IL=0.08\dot{3}$

**Figure 10:**
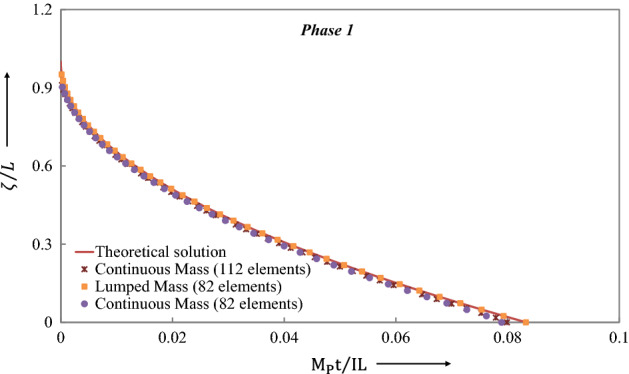
Travel of plastic hinges towards the midspan of the simply supported beam throughout Phase 1predicted by different methods.

Figure 10 shows the evolution of $\xi$, the distance of traveling plastic hinges from midspan, throughout Phase 1. It is observed that the LCP results agree closely with the theoretical solution (Jones^34^) for the first phase of motion.

**Figure 11:**
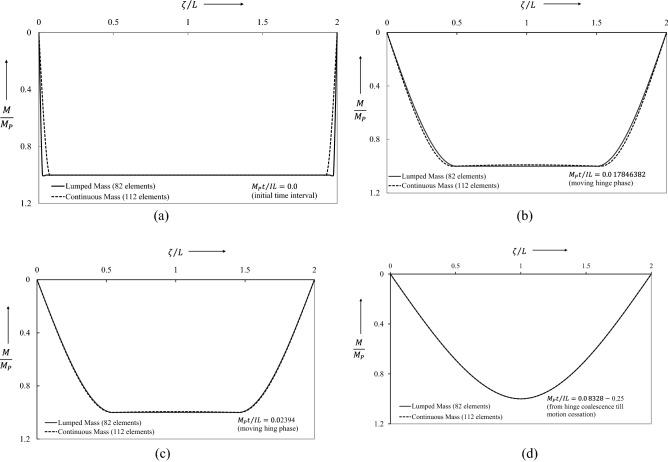
Dynamic bending moment evolution of flexure critical impulsively loaded simply supported beam.

The ensuing dynamic bending moment, initiated from the stationary conditions, is shown in Fig. 11. Figure 11a shows a very small error in the bending moment profile between the lumped mass element and continuous mass elements. For interest, it may be noted that in the previous study^30^ the non-dimensional bending moment showed substantial error at the start, which occurred due to considering an incorrect velocity profile. This error is significantly reduced by using a compatible velocity profile that is generated from proposed the linear program (34). Phase 2 begins at $\overline{t }={M}_{p}t/IL=0.08\dot{3}$ when the traveling plastic hinges meet at midspan. The corresponding bending moment distribution, Fig. 11d, then remains unchanged throughout Phase 2, in which the motion results from the active hinge only at midspan. The motion terminates at $\overline{t }={M}_{p}t/IL=0.25$ when the bending moment distribution jumps to the null state. In contrast to the result obtained from the uniform-mass model, it is seen that the lumped-mass model tracks the changes of the dynamic bending moment accurately throughout the whole motion.

## Effect of transverse shear on simply supported beam

The response of the simply supported beam, Fig. 5, is analyzed by employing finite plastic shear deformation at the support locations. This problem is used as a reference solution to highlight some limitations of the kinetic and kinematic descriptions of (1) and (2) respectively. These descriptions are improved and the numerical solution is compared with the exact solution of Symonds^50^

### Bending-shear material model

**Figure 12:**
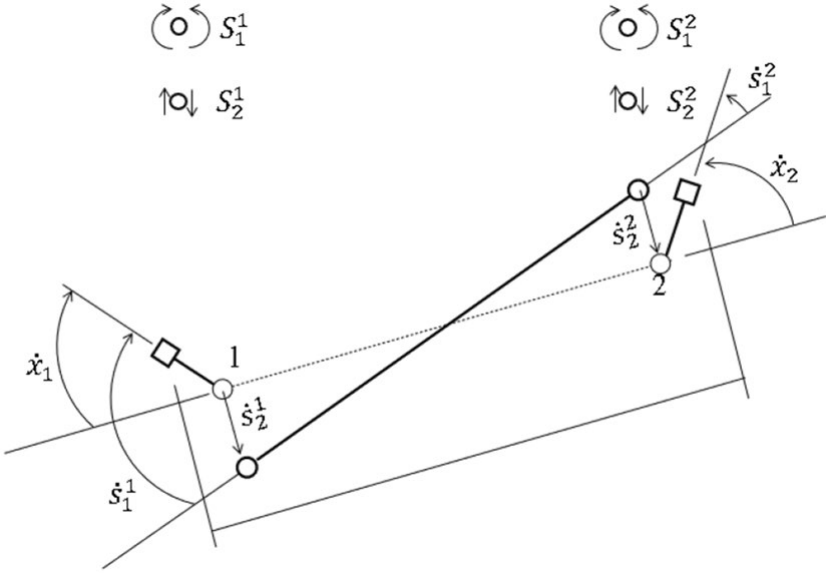
Stress and strain resultants related by the interaction of transverse shear force and bending moment.

A special examination is made of the effect of shear and bending response controlled by the rectangular yield curve. Figure 12 shows the rotational and the shear deformation-rates and their representation by the independent deformation-rates.

The plasticity relation (4) to (7) is still applicable when the shear force interacts with the bending moment, Maier^61^. It is convenient to develop the interactive rectangular yield criterion at a critical section $i$ in a threefold sense:

The yield criterion;

35 $$\left[ {\begin{array}{*{20}c} {y_{*1}^{ + i} } \\ {y_{*1}^{ - i} } \\ {y_{*2}^{ + i} } \\ {y_{*2}^{ - i} } \\ \end{array} } \right] = \left[ {\begin{array}{*{20}c} {X_{*1}^{ + i} } \\ {X_{*1}^{ - i} } \\ {X_{*2}^{ + i} } \\ {X_{*2}^{ - i} } \\ \end{array} } \right] \, - \, \left[ {\begin{array}{*{20}c} { + 1} & 0 \\ { - 1} & 0 \\ 0 & { + 1} \\ 0 & { - 1} \\ \end{array} } \right] \, \left[ {\begin{array}{*{20}c} {M_{i} = S_{{1}}^{i} } \\ {V_{i} = S_{{2}}^{i} } \\ \end{array} } \right];\left[ {\begin{array}{*{20}c} {y_{*1}^{ + i} } \\ {y_{*1}^{ - i} } \\ {y_{*2}^{ + i} } \\ {y_{*2}^{ - i} } \\ \end{array} } \right] \ge \left[ {\begin{array}{*{20}c} 0 \\ 0 \\ 0 \\ 0 \\ \end{array} } \right]$$

$${\mathbf{y}}_{*}^{i} = {\mathbf{X}}_{*}^{i} - {\mathbf{N}}_{i}^{T} \quad {\mathbf{S}}^{i} {\mathbf{y}}_{*}^{i} \ge 0$$

The Normal Flow Rule:

36 $$\left[ {\begin{array}{*{20}c} {\dot{s}_{{1}}^{{\text{i}}} = \dot{\theta }_{i} } \\ {\dot{s}_{{2}}^{{\text{i}}} = \dot{\gamma }_{i} } \\ \end{array} } \right] \, = \, \left[ {\begin{array}{*{20}c} { + 1} & { - 1} & 0 & 0 \\ 0 & 0 & { + 1} & { - 1} \\ \end{array} } \right] \, \left[ {\begin{array}{*{20}c} {\dot{x}_{*1}^{ + i} } \\ {\dot{x}_{*1}^{ - i} } \\ {\dot{x}_{*2}^{ + i} } \\ {\dot{x}_{*2}^{ - i} } \\ \end{array} } \right];\left[ {\begin{array}{*{20}c} {\dot{x}_{*1}^{ + i} } \\ {\dot{x}_{*1}^{ - i} } \\ {\dot{x}_{*2}^{ + i} } \\ {\dot{x}_{*2}^{ - i} } \\ \end{array} } \right] \ge \left[ {\begin{array}{*{20}c} 0 \\ 0 \\ 0 \\ 0 \\ \end{array} } \right];$$

$${\dot{\mathbf{s}}}^{i}={\mathbf{N}}_{i}{\dot{\mathbf{x}}}_{*}^{i}{\dot{\mathbf{x}}}_{*}^{i}\ge 0$$

The Association Rule:

37 $$\left[ {\begin{array}{*{20}c} {y_{*1}^{ + i} } \\ {y_{*1}^{ - i} } \\ {y_{*2}^{ + i} } \\ {y_{*2}^{ - i} } \\ \end{array} } \right]^{T} \, \left[ {\begin{array}{*{20}c} {\dot{x}_{*1}^{ + i} } \\ {\dot{x}_{*1}^{ - i} } \\ {\dot{x}_{*2}^{ + i} } \\ {\dot{x}_{*2}^{ - i} } \\ \end{array} } \right] \, = \, 0$$

$${({\mathbf{y}}_{*}^{i})}^{T}{\dot{\mathbf{x}}}_{*}^{i}=0$$

**Figure 13:**
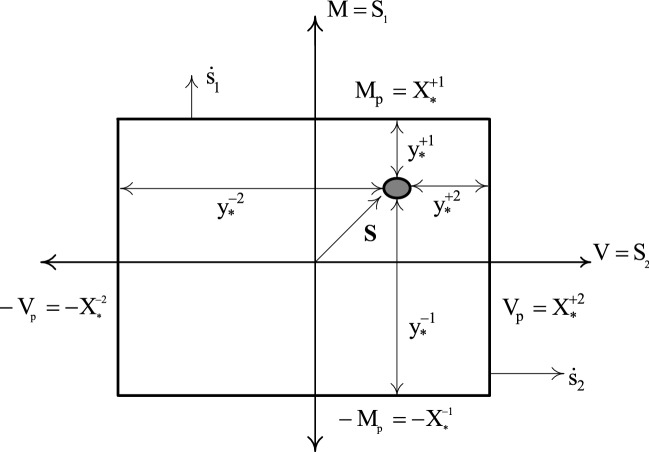
Rectangular yield surface showing the interaction of shear force and bending moment at shear critical section.

Figure 13 relates the bending moment ${S}_{1}$ and the shear force ${S}_{2}$ , at a single critical section $i$, using an approximate rectangular yield curve. The simplicity of the rectangular yield criterion is that, if only ${y}_{*1}^{+i}=0$, a simple plastic hinge having a positive value of ${\dot{\theta }}_{i}$ is activated, and if only ${y}_{*2}^{-i}=0$, a shear slide having a negative value of ${\dot{\gamma }}_{i}$ is activated. Interaction occurs only at a corner of the yield surface, such as ${y}_{*1}^{+i}=0, {y}_{2}^{-i}=0,$ for which plastic deformation may occur simultaneously at the section in a plastic hinge with positive ${\dot{\theta }}_{i}$ and a shear slide with negative ${\dot{\gamma }}_{i}$ .

### Representation of kinetics and kinematics as the nodal description

If the total number of plastic rotations and shear deformations that can be involved in the motion of the beam is $S$, then the kinematic indeterminacy of the general velocity profile is $\beta =S-\alpha$. Unlike the previous investigation^30^, it may happen that $\beta$ is greater than the number of independent velocities associated with the motion of the nodes of the structural model, as may now be demonstrated. Surely, this investigation extends the previous study^30^ toward a more general description of kinetics and kinematics.

Equations (8), (9) need some modification to link the bending and the shear forces and corresponding strain resultant rates $\mathbf{S}$ and $\dot{\mathbf{s}}$ to the independent element forces and deformation rates $\mathbf{X}$ and $\dot{\mathbf{x}}$. For the element, with critical Sects. 1 and 2, as in Figs. 1 and 12, or, more generally, for element $m$ containing critical sections $i$ and $j$, the contributions of this element to the assembly of all elements in Eqs. (8), (9) are

38,39 $$\left[ {\begin{array}{*{20}c} {\dot{x}_{{1}} } \\ {\dot{x}_{{2}} } \\ \end{array} } \right] = \left[ {\begin{array}{*{20}c} 1 & { - 1/\ell } & 0 & { - 1/\ell } \\ 0 & {1/\ell } & 1 & {1/\ell } \\ \end{array} } \right]\left[ {\begin{array}{*{20}c} {\dot{s}_{{1}}^{{1}} } \\ {\dot{s}_{{2}}^{{1}} } \\ {\dot{s}_{{1}}^{{2}} } \\ {\dot{s}_{{2}}^{{2}} } \\ \end{array} } \right]{ ; }\left[ { \, \begin{array}{*{20}c} {S_{{1}}^{{1}} } \\ {S_{{2}}^{{1}} } \\ {S_{{1}}^{{2}} } \\ {S_{{2}}^{{2}} } \\ \end{array} } \right] = \left[ {\begin{array}{*{20}c} 1 & 0 \\ { - 1/\ell } & {1/\ell } \\ 0 & 1 \\ { - 1/\ell } & {1/\ell } \\ \end{array} } \right]\left[ {\begin{array}{*{20}c} {X_{{1}} } \\ {X_{{2}} } \\ \end{array} } \right]$$

$${\dot{\mathbf{x}}}_{m} = \left[ {\begin{array}{*{20}c} {{\mathbf{T}}_{i} } & {{\mathbf{T}}_{j} } \\ \end{array} } \right]\left[ {\begin{array}{*{20}c} {\dot{\mathbf{s}}}^{i} \\ {\dot{{\mathbf{s}}}^{j} } \\ \end{array} } \right]\quad \left[ {\begin{array}{*{20}c} {{\mathbf{S}}^{i} } \\ {{\mathbf{S}}^{j} } \\ \end{array} } \right] = \left[ {\begin{array}{*{20}c} {{\mathbf{T}}_{i}^{T} } \\ {{\mathbf{T}}_{j}^{T} } \\ \end{array} } \right]{\text{X}}_{m}$$

Thus, from (36) and (38), the matrix product $\mathbf{T}\mathbf{N}$ for the whole element assembly is obtained.

If all system nodes are constrained not to move, then ${\dot{\mathbf{x}}}_{m}=0$ for all elements. Despite this, the $n=4$ plastic strain-rates for element $m$, ${\dot{\mathbf{s}}}^{i}$ and ${\dot{\mathbf{s}}}^{j}$ in (38), can still be chosen with some freedom to permit rigid-body motion of that element. The rank of the matrix in (38) is $r=2$, and the number of degrees of freedom in the rigid-body motion is given by ${\beta }_{m}=n-r=2$. If an element has fewer than $n = 4$ possible strain-rates, and the relevant matrix column(s) are deleted, then the rank and $\beta_{m}$ can be similarly determined. It follows that $\beta = \beta_{N} + \beta_{M}$, where $\beta_{N}$ is the number of independent velocities of the nodes, those velocities being the components of $\mathop {{ }{\mathbf{q}}}\limits_{N}$, and $\beta_{M}$ is the number of independent velocities in the elements permitting internal rigid-body motions, those velocities being the components of ${\dot{\mathbf{q}}}_{M}$.

Now, the vector of strain-rates ${\dot{\mathbf{s}}}$ at all critical sections can be expressed in terms of the complete set of $\beta$ independent velocities ${\dot{\mathbf{q}}}$. Thus, ${\dot{\mathbf{x}}}$ in (1) can be replaced by ${\dot{\mathbf{s}}}$,

40 $$\left[ {\begin{array}{*{20}c} {{\dot{\mathbf{s}}}} \\ {{\dot{\mathbf{u}}}} \\ {{\dot{\mathbf{\delta }}}} \\ \end{array} } \right] = \left[ {\begin{array}{*{20}c} {{\mathbf{A}}_{sN} } & {{\mathbf{A}}_{sM} } \\ {{\mathbf{A}}_{dN} } & {{\mathbf{A}}_{dM} } \\ {{\mathbf{A}}_{0N} } & {{\mathbf{A}}_{0M} } \\ \end{array} } \right]\left[ {\begin{array}{*{20}c} {{\dot{\mathbf{q}}}_{N} } \\ {{\dot{\mathbf{q}}}_{M} } \\ \end{array} } \right],$$

and so, by kinetic-kinematic duality, ${\mathbf{X}}$ in the $\beta = \beta_{N} + \beta_{M}$ Eq. (2) can be replaced by ${\mathbf{S}}$,

41 $${\mathbf{Q}} = 0 = {\mathbf{A}}_{s}^{T} {\mathbf{S}} - {\mathbf{A}}_{d}^{T} {{\varvec{\upmu}}} - {\mathbf{A}}_{0}^{T} {{\varvec{\uplambda}}} = \left[ {\begin{array}{*{20}c} {{\mathbf{Q}}_{N} } \\ {{\mathbf{Q}}_{M} } \\ \end{array} } \right] = \left[ {\begin{array}{*{20}c} {{\mathbf{A}}_{sN}^{T} } & {{\mathbf{A}}_{dN}^{T} } & {{\mathbf{A}}_{0N}^{T} } \\ {{\mathbf{A}}_{sM}^{T} } & {{\mathbf{A}}_{dM}^{T} } & {{\mathbf{A}}_{0M}^{T} } \\ \end{array} } \right]\left[ {\begin{array}{*{20}c} {\mathbf{S}} \\ { - {{\varvec{\upmu}}}} \\ { - {{\varvec{\uplambda}}}} \\ \end{array} } \right].$$

Equations (40) and (41) could be used to construct LCP(14) in terms of ${\mathbf{S}}_{n + 1}$. However, reverting from ${\dot{\mathbf{s}}}$ to ${\dot{\mathbf{x}}}$, with ${\mathbf{T}}$ obtained from collecting together (38), for all elements $m$,

42 $${\dot{\mathbf{x}}} = {\mathbf{T\dot{s}}} = \left[ {\begin{array}{*{20}c} {{\mathbf{TA}}_{sN} } & {{\mathbf{TA}}_{sM} } \\ \end{array} } \right]\left[ {\begin{array}{*{20}c} {{\dot{\mathbf{q}}}_{N} } \\ {{\dot{\mathbf{q}}}_{M} } \\ \end{array} } \right] = \left[ {\begin{array}{*{20}c} {\mathbf{A}} & 0 \\ \end{array} } \right]\left[ {\begin{array}{*{20}c} {{\dot{\mathbf{q}}}_{N} } \\ {{\dot{\mathbf{q}}}_{M} } \\ \end{array} } \right],$$

which follows from the fact that ${\dot{\mathbf{x}}} = 0$ whenever ${\dot{\mathbf{q}}}_{N} = 0$. With (42), the second equation set in LCP(14) is uncoupled from the velocities ${\dot{\mathbf{q}}}_{M}$. Also, replacing ${\mathbf{S}}$ by ${\mathbf{X}}$ in (41), using (42),

43 $$\left[ {\begin{array}{*{20}c} {{\mathbf{A}}_{sN}^{T} {\mathbf{T}}^{T} } & {{\mathbf{A}}_{dN}^{T} } & {{\mathbf{A}}_{0N}^{T} } \\ {{\mathbf{A}}_{sM}^{T} {\mathbf{T}}^{T} } & {{\mathbf{A}}_{dM}^{T} } & {{\mathbf{A}}_{0M}^{T} } \\ \end{array} } \right]\left[ {\begin{array}{*{20}c} {\mathbf{X}} \\ { - {{\varvec{\upmu}}}} \\ { - {{\varvec{\uplambda}}}} \\ \end{array} } \right] = \left[ {\begin{array}{*{20}c} {{\mathbf{A}}^{T} } & {{\mathbf{A}}_{dN}^{T} } & {{\mathbf{A}}_{0N}^{T} } \\ 0 & {{\mathbf{A}}_{dM}^{T} } & {{\mathbf{A}}_{0M}^{T} } \\ \end{array} } \right]\left[ {\begin{array}{*{20}c} {\mathbf{X}} \\ { - {{\varvec{\upmu}}}} \\ { - {{\varvec{\uplambda}}}} \\ \end{array} } \right] = \left[ {\begin{array}{*{20}c} 0 \\ 0 \\ \end{array} } \right]$$

In general, when $\beta_{M} > 0$, the first set of equations in LCP(14) consists of $\beta$ equations involving the components of both ${\dot{\mathbf{q}}}_{N}$ and ${\dot{\mathbf{q}}}_{M}$. However, when the masses are lumped at the nodes, submatrix ${\mathbf{A}}_{dM}^{T}$ in (43) is null. When the loads are also applied at the nodes, submatrix ${\mathbf{A}}_{0M}^{T}$ in (43) is also null. Then the first set of equations in LCP(41) is automatically condensed to a set of $\beta_{N}$ equations, uncoupled from the velocities ${\dot{\mathbf{q}}}_{M}$. Automatic condensation does not apply when the masses are distributed in any way within the elements.

### Problem statement

**Figure 14:**
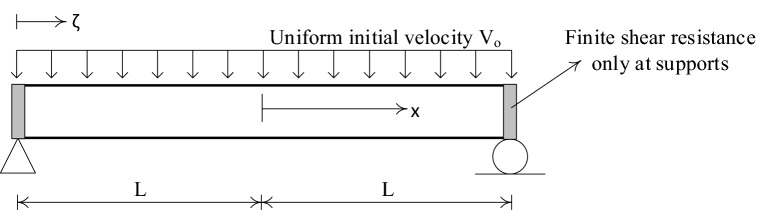
Impulsively loaded simply supported beam including finite shear resistance at the support location.

The problem set out in Fig. 5 is revisited in Fig. 14, but now the beam is assumed to have a finite transverse shear strength $V_{p}$ and plasticity to be governed by the rectangular yield criterion and associated flow rule of Fig. 13. Once more, motion commences with the uniform initial velocity distribution, $V_{0} = I/2mL$.

#### Theoretical solution

A theoretical treatment of a shear flexible simply supported beam of Fig. 14 has been given by Nonaka^64^ and Jones^34^, following an earlier discussion of the problem by Symonds^49^. The character of the solution is much influenced by shear bending ratio

44 $$\nu = V_{p} L/2M_{p}$$

The traveling plastic hinge phase is a suitable problem to illustrate the performance of the LCP solver. According to the theoretical solution, the overall response becomes more bending dominant as $\nu$ increases. For values of $\nu > 1.5$, the theory predicts a motion involving traveling plastic hinges in one of three distinct velocity phases.

**Figure 15:**
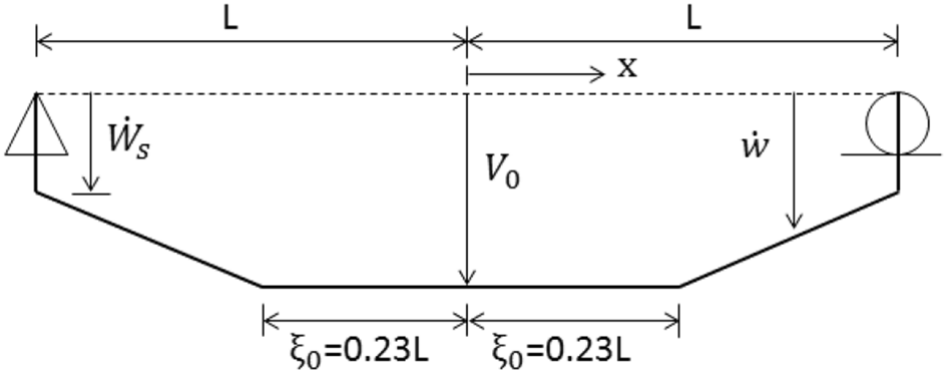
Distribution of transverse velocity during the shear dominant the first phase of motion ($\nu > 1.5$).

The first phase of motion commences with plastic shear deformation at the supports of velocity $\dot{W}_{s}$, together with a pair of stationary plastic bending hinges at location $x = \pm \xi_{0}$, Fig. 15, where $\xi_{0} /L = 1 - 3/2\nu$. It is noted that the choice of $\nu > 1.5$ would make $0 < \xi_{0} < L$. The phase terminates at time $t_{1} = 3mL^{2} V_{0} /\left( {8\nu^{2} M_{P} } \right) = 3IL/\left( {16\nu^{2} M_{P} } \right)$ when $\dot{W}_{s} = 0$ and shear-sliding ceases.

**Figure 16:**
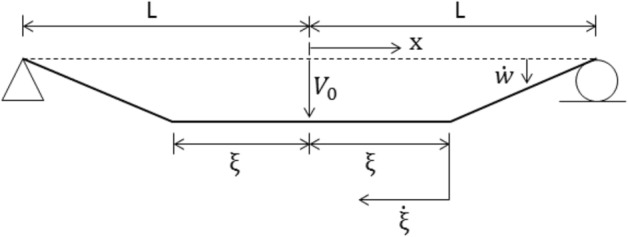
Distribution of transverse velocity during the plastic hinge moving second phase of motion ($\nu > 1.5$).

The second phase of motion initiates when the plastic hinges at $x = \pm \xi_{0}$, start traveling from locations $x = \pm \xi$, Fig. 16, towards midspan, $x = 0$. They arrive at midspan at time $t_{2} = mL^{2} V_{0} /\left( {6M_{P} } \right) = IL/\left( {12M_{P} } \right)$ when the phase ends.

**Figure 17:**
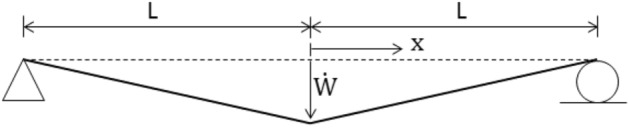
Distribution of transverse velocity during stationary plastic hinge third phase of motion ($\nu > 1.5$).

The third phase of motion then commences when the ensuing deformation is concentrated in a stationary plastic bending hinge at the center of the beam, Fig. 17. This phase terminates at time $t_{3} = mL^{2} V_{0} /\left( {2M_{P} } \right) = IL/\left( {4M_{P} } \right)$ with the residual final central displacement $W_{f} = mL^{2} V_{0} /\left( {3M_{P} } \right) = I^{2} L/\left[ {12\left( {mL} \right)M_{P} } \right]$.

#### LCP Prediction of response

The numerical LCP simulation is conducted using lumped mass elements and uniform mass elements. The beam has been discretized into 100 finite element mesh chosen to capture more faithfully the second phase of motion, the phase of traveling plastic hinges. The strength ratio $\nu = V_{p} L/2M_{p} = 1.72209$ has been back-calculated from the value of $\xi_{0}$.

Table 3 compares the non-dimensional final central displacement $\overline{W}_{f} = (W_{f} /L)\left( {mL} \right)(M_{p} /I^{2} )$ and the non-dimensional time $\overline{t} = M_{p} t/IL$, for the both lumped mass and the uniform mass finite elements. The agreement between the theoretical and LCP results is quite reasonable, particularly when considering the lumped mass discretization.

**Table 3:** Theoretical and LCP results of impulsively loaded beam with bending shear interaction.

	Theoretical solution, Jones^34^	Numerical solution (100 uniform mass elements)	Numerical solution (100 uniform mass elements)	Error (100 lumped mass elements) (%)	Error (100 uniform mass elements) (%)
Final displacement $\overline{W}_{f} = \left( {W_{f} /L} \right)\left( {mL} \right)M_{p} /I^{2}$	0.08333	0.08352	0.08345	-0.22801	-0.14401
Time when shear-sliding ceases $\overline{t}_{1} = M_{p} t_{1} /IL$	0.06322	0.06286	0.06325	0.56944	-0.04745
Time when hinges coalesce $\overline{t}_{2} = M_{p} t_{2} /IL$	0.08333	0.08020	0.08198	-0.07200	2.65211
Cessation time $\overline{t}_{3} = M_{p} t_{3} /IL$	0.25	0.25	0.25	0	0

**Figure 18:**
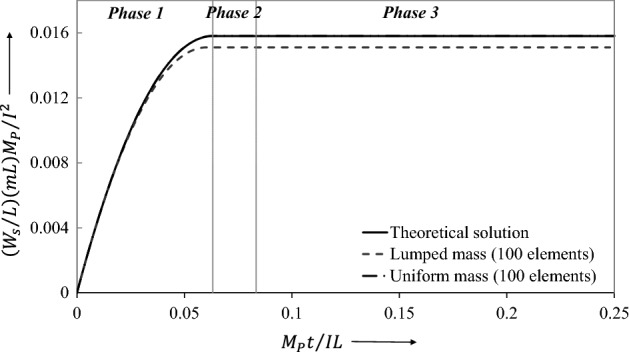
Comparison of transverse shear deformation evolving during the first phase of motion and staying constant in the later phases.

**Figure 19:**
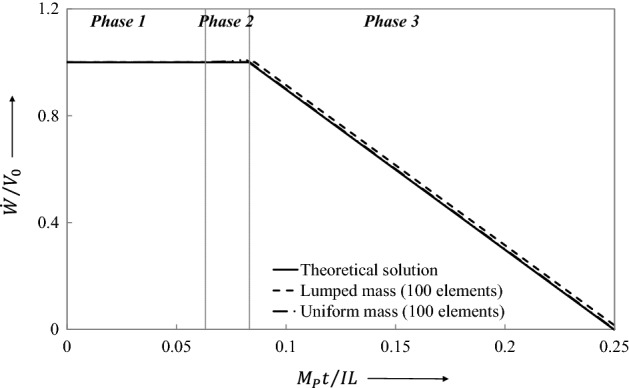
The variation of transverse central velocity, constant in Phase 1 and Phase 2 of motion, and plummeting in Phase 3.

**Figure 20:**
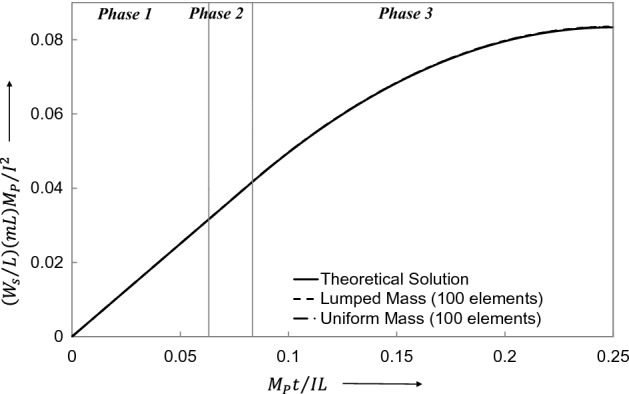
Comparison of beam central displacement changing over time from the start till the standstill.

**Figure 21:**
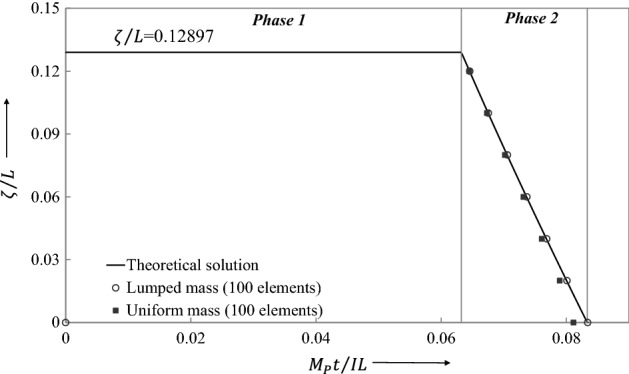
The plastic hinge movement towards the center of the impulsively loaded beam. The theoretical and LCP analysis is compared.

Figures 18, 19, 20, 21 show graphically the evolutionary development in time $\overline{t}$ of the shear deformation $\overline{W}_{s}$, the central transverse velocity $\mathop {\overline{W}}\limits$, the central transverse displacement $\overline{W}$, and the position $\overline{\xi }$ from midspan of the traveling hinges, all in non-dimensional form. Each figure shows the general ability of the LCP method to seek out automatically and follow the changing velocity phases over the whole extent of the motion. In Fig. 19, it will be observed that the lumped elements LCP solution for the transverse central velocity wanders slightly off track when the process is changing from the Phase 2 velocity profile to that of Phase 3. For the uniform mass LCP solution, this difference is hardly visible. In Fig. 21, the LCP solutions identify the position of the stationary plastic hinges in Phase 1 as $\overline{\xi }_{0} = \xi_{0} /L = 0.12897$ and give the end time $\overline{t}_{1}$ of the phase with good accuracy (Table 3). This figure then shows the effective tracking by the LCP solutions of the position $\overline{\xi }$ of these hinges as they travel towards midspan in Phase 2, but it also shows the build-up to a small but slightly more significant error in the predicted end time $\overline{t}_{2}$ of the phase (Table 3).

**Figure 22:**
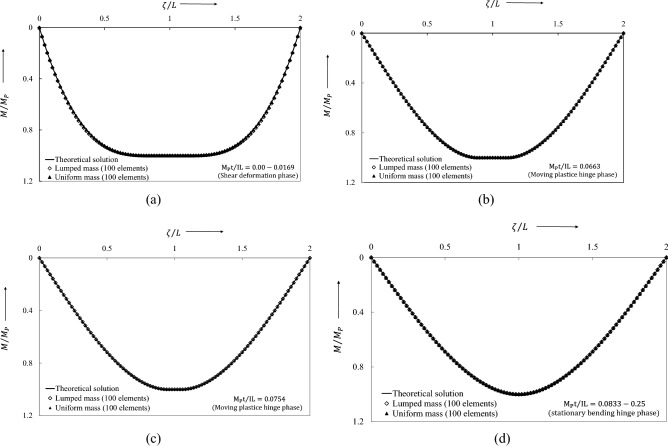
Dynamic bending moment evolution of flexural-shear critical impulsively loaded simply supported beam.

The bending moment distribution along the non-dimensional distance $\zeta /L$ of the beam for $N = 100$ elements is compared with the theoretical result, Fig. 22. Excellent agreement is observed for both choices of discretization, unlike the previous distribution in Fig. 11a. As in the previous case, the motion ceases at the time $\overline{t} = M_{p} t/IL = 0.25$.

## Remarks on rigid plastic analysis and Lemke’s Algorithm

It may be needless to say that the rigid-plastic dynamic analysis is adequately expressive of the true behavior of the concrete and the steel structures, provided that the total input energy transmitted to the structure is significantly larger than the maximum stored elastic strain energy. By excluding all the elastic deformation, rigid-plastic dynamics allow gaining insights into the precise mechanisms responsible for dissipating the plastic energy. The rigid-plastic theory constitutes an intuitive appreciation of plastic deformation, whereas the elasto-plastic theory, although embracing all relevant parameters, tends to define less sharply the evolution of the plastic deformation mechanism. Due to numerical instability, not many computational procedures are available that can determine an intuitive appreciation of the evolutive dynamic rigid-plastic deformation. Most of the commercially available finite element analysis software, although embracing all relevant parameters, tends to offer the evolution of the plastic deformation that is smeared by elasticity. In this context, the current study is concerned with adjusting and improving the previously developed systematic numerical procedure in the form of a linear complementarity problem (LCP)^30^ for the impulsively loaded rigid-plastic beam problems.

In the case of an impulsively loaded beam subdivided into continuous mass elements having infinite resistance to shear deformation, the shear force causes the initial velocity profile to zig-zag like a tooth profile of a saw. Such a profile happens to be taxing on the LCP solver presenting considerable numerical difficulties, particularly with larger discretization. A workaround proposed previously^30^ to overcome this difficulty was to use the lumped mass velocity profile instead. The proposed linear program (34) manages to overcome this intrinsic limitation of the LCP approach by providing a geometrically compatible velocity for the stable implementation of the algorithm. This implementation of the linear program in the Lemke solver^65–68^ has proven to be a much more robust technique for simulating refined beam discretization.

Nevertheless, numerical instability issues re-emerged in the second investigation of the impulsively loaded simply supported beam in which the dynamic response involves shear deformation at the simple supports and bending deformation elsewhere. The previous description of kinetics (1) and kinematics (2) suffered irregular convergence when applied to the bending-shear interaction problem due to unconstrained rigid body motion. Therefore, modification is made to the kinetic and kinematic description, shown in (40) and (41) respectively. This strategy helped to significantly improve the convergence of the LCP solver.

## Conclusions

This study is concerned with adjusting and improving the previously developed numerical procedure in the form of a linear complementarity problem (LCP)^30^. Numerical difficulties concerning the previously investigated impact problem^30^ can be summarized as follows:

- The use of the actual initial velocity profile in the continuously discretized beam can lead to severe numerical difficulties and inaccurate prediction of the impacted beam having infinite resistance to shear deformation
- Rigorous models involving bending-shear interaction can lead to numerical instability issues if the kinematic descriptions are not defined in terms of independent velocities of the nodes and independent velocities permitting the internal rigid body motions.

In the context of the above limitations, extra support is provided to the LCP formulation to make it computationally robust. Thus, a linear program (LP) is developed that determines an optimized initial velocity profile, which is necessary for initiating the dynamic response. For illustrative purposes, the improved LCP formulation is applied to an example of an impulsively loaded simply supported beam having finite bending strength and infinite shear strength. In comparison with the available continuum solution, the LCP analysis generates the complete evolution of all response parameters with reasonably good accuracy. However, the LCP solver becomes susceptible to breakdowns when a second illustrative numerical test is performed on the same beam, but with finite shear resistance at supports. Enhancing the kinetic and kinematic description to permit the internal rigid body motion renders the LCP solver much more robust and overcomes all the numerical difficulties. The numerical results are again compared to the continuum solution of the interaction problem borrowed from literature. The accuracy and computational efficiency of the LCP bending shear interaction model are demonstrated by accurate prediction of the bending moments, deflections, and failure mechanisms. In this regard, it is worth stressing, that the scope of this enhanced formulation can be widened to include the interaction effects of torsion and axial force.

## Data Availability

The authors declared that “All data generated or analysed during this study are included in this published article”.
